# Management of Pediatric Mild Traumatic Brain Injury Patients: S100b, Glial Fibrillary Acidic Protein, and Heart Fatty-Acid-Binding Protein Promising Biomarkers

**DOI:** 10.1089/neur.2024.0027

**Published:** 2024-05-31

**Authors:** Anne-Cécile Chiollaz, Virginie Pouillard, Fabian Spigariol, Fabrizio Romano, Michelle Seiler, Céline Ritter Schenk, Christian Korff, Céline Habre, Fabienne Maréchal, Verena Wyss, Lyssia Gruaz, Marcel Lamana-Vallverdu, Elvira Chocano, Lluis Sempere Bordes, Carlos Luaces-Cubells, María Méndez-Hernández, José Antonio Alonso Cadenas, María José Carpio Linde, Paula de la Torre Sanchez, BIOTRABIS investigators and participants, t-BIOMAP investigators and participants,, Joan Montaner, Sergio Manzano, Jean-Charles Sanchez

**Affiliations:** ^1^ Internal Medicine Department, Faculty of Medicine, University of Geneva, Geneva, Switzerland.; ^2^ Pediatric Neurology Unit, Woman, Child and Adolescent Department, Geneva University Hospitals, Geneva, Switzerland.; ^3^ Pediatric Emergency Department, Neuchâtel Hospital (RHNE), Neuchatel, Switzerland.; ^4^ Division of Pediatric Emergency Medicine, Department of Pediatrics, Inselspital, Bern University Hospital, University of Bern, Switzerland.; ^5^ Pediatric Emergency Department, University Children's Hospital Zurich, Zurich, Switzerland.; ^6^ Department of Pediatrics, Fribourg Hospital HFR, Fribourg, Switzerland.; ^7^ Division of Radiology, University Hospitals of Geneva, Geneva, Switzerland.; ^8^ Platform of Pediatric Clinical Research, Woman, Child and Adolescent Department, Geneva University Hospitals, Geneva, Switzerland.; ^9^ Neurovascular Research Laboratory, Vall d'Hebron Institute of Research, Autonomous University of Barcelona, Barcelona, Spain.; ^10^ Neurovascular Research Group, Institute of Biomedicine in Sevilla, Sevilla, Spain.; ^11^ Pediatric Emergency Service, University Hospital San Joan de Deu, Esplugues del Llobregat, Barcelona, Spain.; ^12^ Pediatric Service, University Hospital Germans Trias i Pujol, Barcelona, Spain.; ^13^ Pediatric Department, University Hospital Niño Jesús, Madrid, Spain.; ^14^ Pediatric Emergency Service, University Hospital Virgen de la Macarena, Sevilla, Spain.; ^15^ Pediatric Emergency Department, Geneva University Hospitals and Faculty of Medicine, University of Geneva, Geneva, Switzerland.

**Keywords:** biomarkers, diagnostics, emergency, mild traumatic brain injury (mTBI), pediatric, triage

## Abstract

Children are highly vulnerable to mild traumatic brain injury (mTBI). Blood biomarkers can help in their management. This study evaluated the performances of biomarkers, in discriminating between children with mTBI who had intracranial injuries (ICIs) on computed tomography (CT+) and (1) patients without ICI (CT−) or (2) both CT− and in-hospital-observation without CT patients. The aim was to rule out the need of unnecessary CT scans and decrease the length of stay in observation in the emergency department (ED). Newborns to teenagers (≤16 years old) with mTBI (Glasgow Coma Scale > 13) were included. S100b, glial fibrillary acidic protein (GFAP), and heart fatty-acid-binding protein (HFABP) performances to identify patients without ICI were evaluated through receiver operating characteristic curves, where sensitivity was set at 100%. A total of 222 mTBI children sampled within 6 h since their trauma were reported. Nineteen percent (*n* = 43/222) underwent CT scan examination, whereas the others (*n* = 179/222) were kept in observation at the ED. Sixteen percent (*n* = 7/43) of the children who underwent a CT scan had ICI, corresponding to 3% of all mTBI-included patients. When sensibility (SE) was set at 100% to exclude all patients with ICI, GFAP yielded 39% specificity (SP), HFABP 37%, and S100b 34% to rule out the need of CT scans. These biomarkers were even more performant: 52% SP for GFAP, 41% for HFABP, and 39% for S100b, when discriminating CT+ versus both in-hospital-observation and CT− patients. These markers can significantly help in the management of patients in the ED, avoiding unnecessary CT scans, and reducing length of stay for children and their families.

## Introduction

Traumatic brain injury (TBI) can affect anyone, with children being particularly vulnerable. The worldwide incidence of pediatric TBI ranges between 47 and 280 per 100,000 children.^1^ The high majority (80%) are defined as mild TBI (mTBI) if it includes (1) one or more of the following: confusion or disorientation, loss of consciousness (LOC) for up to 30 min, post-traumatic amnesia for up to 24 h, and/or other transient neurological abnormality; and (2) Glasgow Coma Scale (GCS) score of 13–15.^1–3^ TBI is induced by an external blow or jolt to the head, including a rapid movement of acceleration/deceleration, resulting in a transfer of mechanical energy into the brain. This can lead to intracranial injuries (ICIs) such as hemorrhage that may require surgical intervention.^1^ Prompt detection and management of ICI are critical, as complications can evolve quickly and can lead to disability or injury-related death. In these circumstances, the first objective for clinicians is to detect all patients in need of neurosurgical intervention after TBI. ICIs are typically diagnosed using cranial computed tomography (CT) scan. However, children are particularly at risk of cancer secondary to irradiation,^4–7^ and care should be taken to avoid unnecessary exposure to ionizing radiation. Indeed, only a very small proportion presents clinically important injuries on CT, with up to 90% having mTBI associated with negative imaging.^2^ Clinicians can follow Pediatric Emergency Care Applied Research Network (PECARN) recommendations^8^ to identify children with very low risk for clinically important TBI (ciTBI) following head trauma, who would not require imaging. In Europe and the United States, CT is performed in only 10–35% of mTBI cases.^7,9^ Most of the children are therefore kept under observation in the emergency department (ED) to monitor their symptoms for 6–24 h. This observation time is, however, stressful for children and parents and cost-consuming for the health care system. An improved management of children suffering from mTBI is thus needed in the ED.

To address these challenges, the study of protein biomarkers released in the blood by cells of the neurovascular unit after mTBI might provide objective information to guide patient triage and management. In adults, several proteins have been investigated as stratification markers in mTBI. These include S100 calcium-binding protein B (S100b),^10^ glial fibrillary acidic protein (GFAP), both astrocyte proteins,^11^ and heart fatty-acid-binding protein (HFABP), an intracellular vascular and neuronal fatty-acid transporter.^12,13^ Despite extensive research on these proteins in adults, and even the introduction of S100b measurement in the Scandinavian^14–16^ and more recently French^17^ guidelines, there is limited knowledge regarding their relevance in the pediatric population.^18–21^

Our aim is to assess the performances of these biomarkers in a prospective multicenter pediatric cohort of patients, to predict the absence of ICI with high sensitivity and avoid both, unnecessary CT scans and long stay in the ED.

## Materials and Methods

### Study population

Children were recruited in two prospective multicenter cohort studies that took place in five pediatric EDs in Switzerland between October 2020 and February 2023 and in four pediatric EDs in Spain between 2019 and 2021. Both studies received institutional review board approval and were conducted in accordance with Good Clinical Practice guidelines and provisions of the Declaration of Helsinki. Both studies are registered in www.clinicaltrials.gov: NCT06233851 and NCT04641767. Informed consent was obtained from all subjects involved in the study or from their representatives.

We included any child of 16 years or younger presenting at the ED with a head trauma within <24 h with, in addition, (1) the GCS of 14; or (2) GCS of 15 and one of the following symptoms: LOC for <30 min with onset within the first 20 min post-trauma, post-traumatic amnesia of <24 h duration with onset within 30 minu post-trauma, persistent headaches, irritability, three or more episodes of vomiting, confusion, dizziness, or transient neurological abnormality such as seizure; or (3) signs for basal skull fracture (e.g., hemotympanum, raccoon eyes, cerebrospinal fluid otorrhea or rhinorrhea, Battle’s sign); or (4) high-energy trauma (such as traffic accident or fall from more than 0.9 m [3 ft.] if <2 years old, more than 1.5 m [5 ft.] if ≥2 years old). Exclusion criteria were patients already included in another clinical study with pharmacologic treatment, alcohol consumption or use of other substances, history of recent TBI (within the last month prior consultation), recent history of epileptic seizures (within the last month prior consultation), Down syndrome, acute encephalopathy, encephalitis, or meningitis. A control group was also recruited. Inclusion criteria were any child of 16 years or younger with scheduled blood sample in the ambulatory care unit and without TBI. Exclusion criteria were the same as defined for the TBI group.

The attending physician obtained written consent from the parents or legal guardians of children meeting the inclusion criteria for participation in the study as well as the children themselves if they were older than 14 years. Blood was drawn as soon as possible, no later than 24 h after the trauma. The study did not interfere in any medical decision such as to perform a CT scan or not, to place the patient in observation, or to do any other blood test.

Study data were collected and managed using REDCap electronic data capture tools hosted at Hôpitaux Universitaires de Genève (HUG).^22,23^

### CT scan analysis

All CT scans were reviewed by the same pediatric radiologist (C.H.) blinded for the clinical evaluation, biomarker result, and local CT conclusion. CT scan was considered positive with ICI defined by the presence of any of these findings: intracranial hemorrhage or contusion, cerebral edema, traumatic infarction, diffuse axonal injury, shearing injury, sigmoid sinus thrombosis, midline shift of intracranial contents or signs of brain herniation, diastasis of the skull, pneumocephalus or skull fracture depressed by at least the width of the table of the skull (PECARN criteria^8^).

### Blood biomarker analysis

Serum samples were obtained as soon as possible in the ED. Samples were centrifuged and stored at −80°C. S100b, GFAP, and HFABP were measured by enzyme-linked immunosorbent assay using, respectively, R-plex Human S100b (F212E), GFAP (F211M) and FABP3/HFABP (F214T) Antibody Sets (Meso Scale Diagnostics, Rockville, MD, USA). Lower limit of detection (LLoD) was respectively 1.6 pg/mL with a calibration range of 1.22–5000 pg/mL for S100b, 63 pg/mL and 122–500,000 pg/mL for GFAP, 90 pg/mL and 24.41–100,000 pg/mL for HFABP. Lower limit of quantification (LLoQ) was defined as the lowest concentration with a coefficient of variation (CV) below 20% and a recovery between 80% and 120%. All the kits were used as stated in the manufacturers’ guidance. Duplicate control serum was measured on each plate, and intra- and interplate CVs were below 20%.

### Outcome measures

Primary outcome was the presence of ICI on CT. Diagnostic values of the blood biomarkers GFAP, S100b, and HFABP were evaluated to rule out the need of unnecessary CT scans and decrease the length of stay in observation in ED.

### Statistical analysis

For the analyses, patients were classified according to their status: stayed in observation without CT (in-hospital-observation), CT performed with negative result (CT−), or CT performed with positive result (CT+).

Statistical analysis was performed using R (http://www.rproject.org, version 4.3.0) in RStudio (http://www.rstudio.com, version 2023.06.0). Biomarker concentrations were normalized using their medians as correction factors. Patients were dichotomized into the following groups: (1) CT− versus CT+ groups or (2) in-hospital-observation without CT and CT− versus CT+ groups. Differences between groups were established using the nonparametric Mann–Whitney *U* test, given that the Kolmogorov–Smirnov test reveals that all proteins were non-normally distributed (*p* < 0.05). Analysis of variance (ANOVA) was used when comparing more than two groups. Chi-squared test was used for statistical analyses of the clinical data. Statistical significance was inferred at *p* < 0.05. The levels of S100b, GFAP, and HFABP are presented through box- and dot-plots, with a log10 Y-scale. Biomarker’s ability for classifying patients according to their CT-result group is evaluated using receiver operating characteristic (ROC) curves with the pROC package in R. Iterative combinations of each two-protein panels were tested with the PanelomiX threshold-based algorithm.^24,25^ For each biomarker and its combination, the optimal performance was investigated to identify all patients without ICI, therefore looking for the highest specificity when sensitivity was set at 100%. Two separated analyses were performed, with blood sampling done within (a) 6 h and (b) 24 h post-trauma (Supplementary Figures S2 and S3).

## Results

**FIG. 1.**
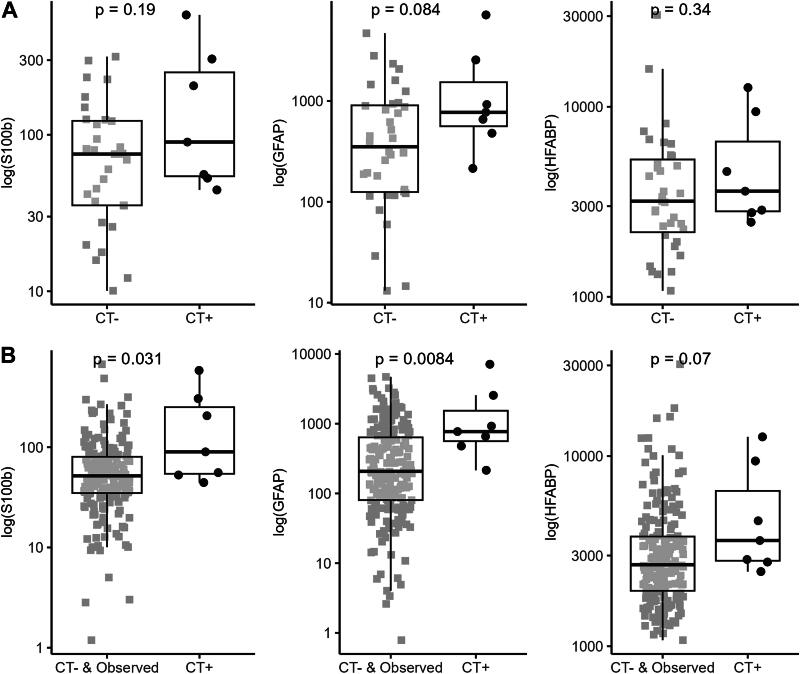
S100b, GFAP, and HFABP serum concentrations in mTBI patients (within 6 h). **(A)** Biomarker expression within CT− and CT+ mTBI patients (only CT-scanned patients). **(B**) Biomarker expression within CT− or in-hospital-observation patients and CT+ mTBI patients (CT-scanned and observed without CT [>6 h at ED] patients). Positive CT is based on PECARN criteria. Box plots represent median and IQR for compared groups; dot plots represent for each patient log-scaled biomarker concentration. The analysis was carried out using a Mann–Whitney *U* test (shown *p* value). CT, computed tomography; GFAP, glial fibrillary acidic protein; HFABP, heart fatty-acid-binding protein; mTBI, mild traumatic brain injury; PECARN, Pediatric Emergency Care Applied Research Network.

**FIG. 2.**
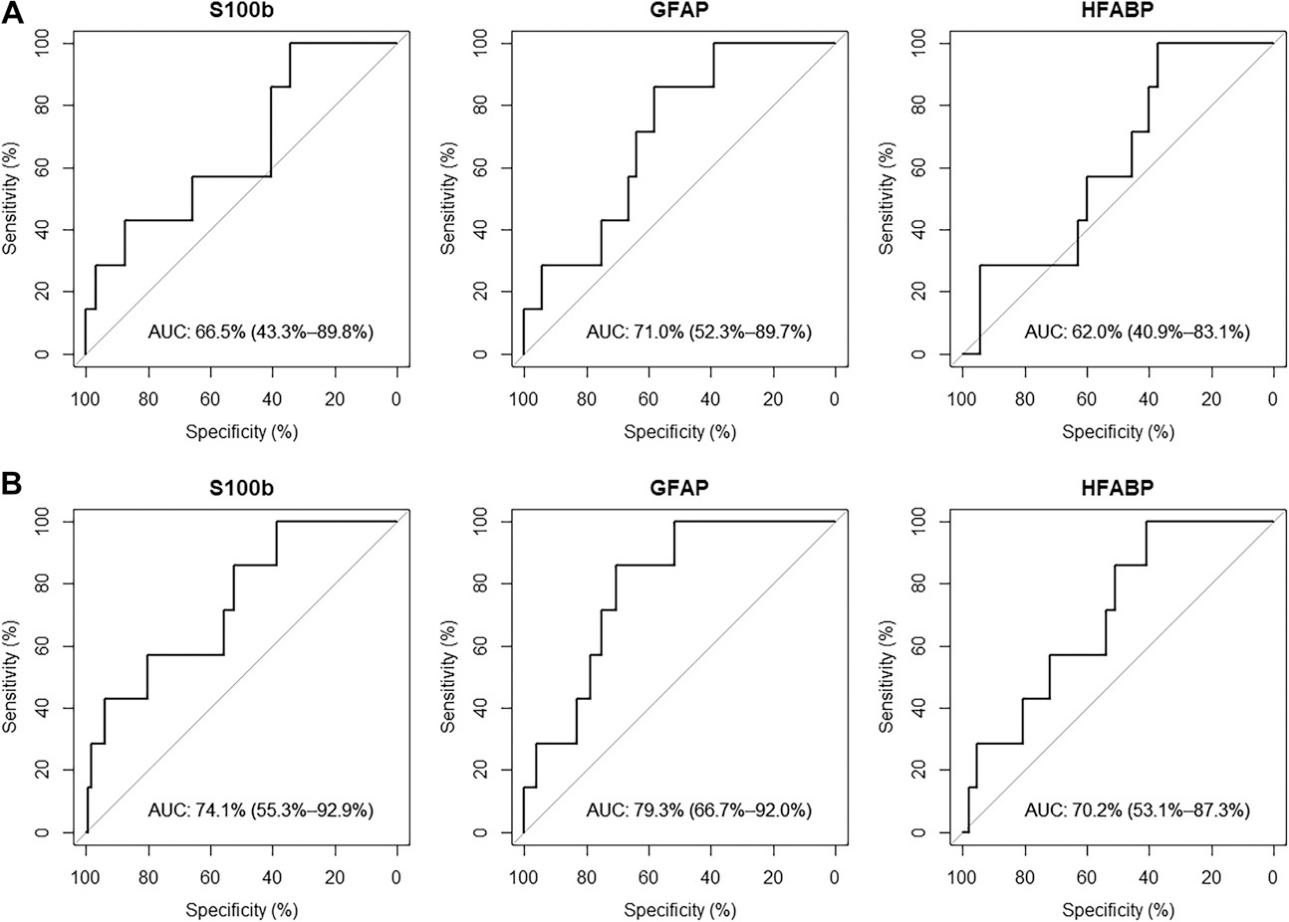
S100b, GFAP, and HFABP diagnostic performances to classify mTBI patients (within 6 hours). **(A**) Diagnostic performances within CT− and CT+ mTBI patients (only CT-scanned patients). **(B**) Diagnostic performances within CT− or in-hospital-observation patients and CT+ mTBI patients (CT-scanned and observed without CT [>6 h at ED] patients). Receiver operating characteristic (ROC) curves in mTBI patients. AUC = area under the curve with 95% confidence interval. Performances were investigated at 100% sensitivity and corresponding highest specificity. CT, computed tomography; GFAP, glial fibrillary acidic protein; HFABP, heart fatty-acid-binding protein; mTBI, mild traumatic brain injury.

A total of 376 serum samples were analyzed. This included 74 control patients without head trauma and 302 mTBI patients. Out of the mTBI patients, 222 patients had blood sampling done within 6 h after their head trauma. A total of 179 patients (81%) did not undergo a CT scan examination but were kept in observation for symptoms monitoring at the ED for more than 6 h (Table 1). Within CT-scanned patients (*n* = 43), seven (16%) were positive (Tables 1 and 2). Mean age was 8 years old in all groups (standard deviation [SD] of 4,93-4,43-5,08-4,93 for respective controls, mTBI without CT, mTBI with CT−, and mTBI with CT+ groups). A wide range of ages from newborn to teenagers was observed (from 1 month to 16 years old). Most of the patients had a GCS of 15 with associated symptoms. LOC, post-traumatic amnesia, persistent headaches, and more than three episodes of vomiting were the most frequent symptoms in mTBI patients (Table 1). Out of the seven patients with a CT+ result, two of them also presented other body fractures, and all of them had simple skull fractures seen on CT scan. CT+ patients often had more than one PECARN criteria that might define a CT+ result. There were five (71%) cases of intracranial hemorrhage (more specifically a subdural hemorrhage), five (71%) pneumocephalus, two (29%) diastasis of the skull, one (14%) midline shift or intracranial contents or signs of brain herniation, and one (14%) skull fracture depressed by at least the width of the table of the skull (Table 2). Except for the presence of fractures, no significant differences in clinical parameters were observed comparing patients with or without ICI on CT or kept in observation without CT (Table 1). Time between head trauma and blood sampling was equivalent within compared groups (median of 4 h in observed and CT− groups, 3 h in CT+ group). Age correlation was investigated in the control group without head trauma. Spearman correlation revealed that both S100b and GFAP were age-inversed correlated in the whole control group, whereas HFABP was not (*r* = −0,43, *p <* 0.0001 for S100b, *r* = −0.71, *p <* 0.0001 for GFAP and *r* = −0.23, *p* > 0.05 for HFABP; Supplementary Fig. S1A). A significant positive correlation between GFAP and S100b was also reported (Supplementary Fig. S1B). Means and standard deviations of S100b, GFAP, and HFABP in each group of patients are reported in Table 1. There were significant differences in biomarker expression comparing groups (ANOVA, *p* < 0.001; Table 1). Blood concentration of the three biomarkers was increased in CT+ patients compared with others and was significantly different for S100b and GFAP when comparing with both CT− and in-hospital-observation patients (Fig. 1A and 1B, and Supplementary Tables S2 and S3). ROC curve analysis, for diagnostic performances of the three biomarkers, allowed to select the best specificity when the sensitivity was set at 100% to exclude all children with ICI (Fig. 2A and 2B). In these conditions, GFAP yielded the best specificity (39% SP) to identify CT− patients with 100% sensitivity, followed by HFABP with 37% SP and S100b with 34% SP (Table 3A). Performances of these three biomarkers were higher to identify both CT− and in-hospital-observation patients, with, respectively, 52%, 41%, and 39% of specificity for GFAP, HFABP, and S100b (Table 3B). All two-protein panels showed increased specificity over the best performing single biomarker, GFAP. The best performing panel was the combination of GFAP and HFABP, which reached 68% specificity (Supplementary Table S3). All the results were based on the analysis of patients sampled within ≤6 h post-trauma. Same analysis on all patients (*n* = 302) sampled within 24 h was performed (Supplementary Tables S4–S8 and Figs. S2–S3). At 100% sensitivity, S100b performed with 32% SP, GFAP with 27% SP, whereas HFABP was neutral to identify CT− or in-hospital-observation patients.

**Table 1.** Clinical Parameters and Biomarker Expression in Controls and mTBI Patients (With or Without CT Scan)—Within 6 H Post-Trauma

	Ctrl *n* = 74	Mild TBI *n* = 222			*p* value
		No CT *n* = 179 (81%)	CT *n* = 43 (19%)		
			CT− *n* = 36 (84%)	CT+ *n* = 7 (16%) (3% of mTBI)	
Age (year old)					
** **Mean (SD)	8.70 (4.93)	8.42 (4.43)	8.00 (5.08)	8.84 (4.93)	0.925
** **Median (Min, Max)	8.75 (0.10, 16.8)	8.60 (0.700, 16.0)	6.80 (0.100, 16.0)	10.1 (0.900, 15.0)	
Sex					
** **Boys	40 (54.1%)	98 (54.7%)	22 (61.1%)	5 (71.4%)	0.735
** **Girls	34 (45.9%)	81 (45.3%)	14 (38.9%)	2 (28.6%)	
Severity of injury, *n* (%)					
** **GCS14	—	12 (6.7%)	8 (22.2%)	3 (42.9%)	<0.001
** **GCS15	—	167 (93.3%)	28 (77.8%)	4 (57.1%)	
Symptoms at inclusion, *n* (%)					
** **Loss of consciousness	—	35 (19.6%)	8 (22.2%)	1 (14.3%)	0.672
** **Post-traumatic amnesia	—	54 (30.2%)	10 (27.8%)	0 (0%)	0.357
** **Persistent headaches	—	50 (27.9%)	9 (25.0%)	1 (14.3%)	0.951
** **More than three episodes of vomiting	—	34 (19.0%)	4 (11.1%)	1 (14.3%)	0.721
** **Vertigo	—	14 (7.8%)	0 (0%)	1 (14.3%)	0.122
** **Confusion	—	20 (11.2%)	7 (19.4%)	0 (0%)	0.135
** **Convulsion	—	3 (1.7%)	2 (5.6%)	0 (0%)	0.221
TBI and others body fractures, *n* (%)					
** **TBI only	—	170 (95.0%)	31 (86.1%)	5 (71.4%)	0.004
** **TBI + other fractures	—	7 (3.9%)	5 (13.9%)	2 (28.6%)	
Skull fracture (on CT), *n* (%)					
** **Yes	—	—	9 (25.0%)	7 (100%)	<0.001
Timelap TBI-blood (h)					
** **Mean (SD)	—	4.28 (1.18)	3.86 (1.60)	3.43 (1.62)	0.123
** **Median (Min, Max)	—	4.00 (1.00, 6.00)	4.00 (1.00, 6.00)	3.00 (2.00, 6.00)	
S100b (pg/mL)					
** **Mean (SD)	39.3 (38.7)	67.2 (74.2)	92.4 (80.5)	191 (198)	<0.001
** **Median (Min, Max)	27.9 (1.79, 220)	51.0 (1.19, 672)	75.3 (10.1, 316)	89.9 (44.4, 583)	
** **Missing	11 (14.9%)	14 (7.8%)	4 (11.1%)	0 (0%)	
GFAP (pg/mL)					
** **Mean (SD)	97.6 (141)	471 (679)	737 (967)	1820 (2460)	<0.001
** **Median (Min, Max)	64.0 (3.37, 951)	198 (0.787, 4480)	354 (13.2, 4690)	771 (215, 7120)	
** **Missing	4 (5.4%)	2 (1.1%)	0 (0%)	0 (0%)	
HFABP (pg/mL)					
** **Mean (SD)	2270 (3830)	3430 (2590)	4710 (5280)	5470 (3960)	<0.001
** **Median (Min, Max)	1580 (790, 33,600)	2640 (1150, 17,900)	3190 (1080, 30,400)	3600 (2470, 12,600)	
** **Missing	1 (1.4%)	4 (2.2%)	1 (2.8%)	0 (0%)	

CT, computed tomography; Ctrl, control; GCS, Glasgow Coma Scale; Max, maximum; Min, minimum; mTBI, mild traumatic brain injury; SD, standard deviation.

**Table 2.** PECARN Criteria for Positive CT

	CT+ (*n* = 7)
1. Intracranial hemorrhage or contusion	5 (71.4%)
Subarachnoid hemorrhage	0 (0%)
Epidural hemorrhage	2 (28.6%)
Intraparenchymal hemorrhage	1 (14.3%)
Subdural hemorrhage	5 (71.4%)
2. Cerebral edema	0 (0%)
3. Traumatic infraction	0 (0%)
4. Diffuse axonal injury or shearing injury	0 (0%)
5. Sigmoïd sinus thrombosis	0 (0%)
6. Midline shift of intracranial contents or signs of brain herniation	1 (14.3%)
7. Diastasis of the skull	2 (28.6%)
8. Pneumocephalus	5 (71.4%)
9. Skull fracture depressed by at least the width of the table of the skull	1 (14.3%)

CT, computed tomography; PECARN, Pediatric Emergency Care Applied Research Network.

**Table 3.** S100b, GFAP, and HFABP Best Performances to Rule Out mTBI Patients (Within 6 H)

Biomarker	Sensitivity (%)	Specificity (%)	AUC (%)	Threshold (pg/mL)
(A)				
S100b	100	34.38	66.52	43.06
GFAP	100	38.89	71.03	204.22
HFABP	100	37.14	62.04	2456.57
(B)				
S100b	100	38.58	74.11	44.34
GFAP	100	51.64	79.34	214.02
HFABP	100	40.95	70.20	2456.57

(A) Best performances to rule out a maximum of CT− patients, whereas all CT+ patients have been identified (only CT-scanned patients). GFAP yields the best performances (100% SE–39% SP).

(B) Best performances to rule out a maximum CT− and in-hospital-observation patients, whereas all CT+ patients have been identified (CT-scanned and observed without CT [>6 h at ED] patients). GFAP yields the best performances (100% SE–52% SP).

AUC, area under the curve; ED, emergency department; GFAP, glial fibrillary acidic protein; HFABP, heart fatty-acid-binding protein; mTBI, mild traumatic brain injury; SE, sensibility; SP, specificity.

## Discussion

This pediatric study on mTBI investigated the performances of three blood biomarkers in discriminating both CT− and in-hospital-observation without CT patients versus CT+ patients. The incidence of ICI was of 3%, which is in accordance with the literature.^1,3^ Blood measurements of GFAP, HFABP, and S100b within 6 h post-trauma could identify 39%, 37%, and 34% of children with CT− result, respectively, whereas all children with ICI on CT were excluded. These results confirm what Manzano et al. have shown in a previous pediatric cohort of mTBI patients, where S100b was a valuable tool to help the physician to decide whether head CT was indicated with 100% sensitivity and 34% of specificity.^9,26^

Physicians need to balance radiation exposure with the need to not miss any serious head trauma in children. However, in children, concerns should not be limited to the patient undergoing CT scan after mTBI. We report 19% of CT scan examinations, meaning that in fact most of the children do not undergo CT scan examination but stayed in observation in the ED (81%). The CT acquisition rate in children is lower than in adults. Kupperman et al. reported 35% of CT scan examinations^8^ and identified a group of very low-risk ci TBI for whom they suggest observing in the ED to monitor symptom evolution instead of performing a CT scan. Monitoring the occurrence of bleeding usually involves an observation period that generally extends from 6 to 24 h in the hospital. This time is stressful for children and parents, and time- and cost-consuming for the hospital. The clinical management of pediatric mTBI patients therefore differs compared with the adult one. Clinical studies in pediatric mTBI biomarkers must not only attempt to identify CT− patients but also to identify patients kept in observation for hours in the ED (in-hospital-observation patients). In our study, GFAP, HFABP, and S100b measured within 6 h after the trauma yielded up to 52%, 41%, and 39% of specificity to identify both CT− and in-hospital-observation children, respectively.

S100b currently stands as the most extensively studied biomarker and might be used in the acute management of pediatric mTBI patients, with one-third of patients for whom an unnecessary CT scan could be avoided.^10^ However, the false-positive rate is still too high and precludes its use in clinical practice. Our results corroborated S100b performances while also highlighting the superior potential of two other biomarkers GFAP and HFABP.

Furthermore, Bouvier et al. showed that S100b was inversely correlated with age in children and identified at least three age categories.^27^ These findings emphasize the difficulties in the future use of S100b and its defined thresholds in routine clinical practice regarding patient’s age. Our study covered the wide range of age, from newborns to teenagers, and all the compared groups had the same mean age of 8 years. We also reported a significant age correlation for both S100b and GFAP in the control cohort of children without head trauma. Age stratification to define thresholds could potentially be needed in further studies while using markers such as S100b or GFAP. HFABP, on the contrary, did not show any age correlation. This lack of age dependency gives HFABP a distinct advantage, making it a potentially more convenient biomarker for clinical use across all pediatric patients.

Performances of biomarkers could be affected by the sampling time. Previous studies on S100b have shown its short half-life in blood and recommend a maximum delay of 3–6 h for sampling after the head trauma.^14,15,17,26,28^ GFAP increases more slowly after a head trauma and peaks at 20 h.^29^ HFABP as a marker of brain damage in stroke has been seen to peak already at 2–3 h and to remain elevated up to 120 h.^30^ In our cohort, we did two separated analyses of performances, depending on sampling time after the trauma (within 6 h and 24 h). S100b and GFAP remained significantly different between in-hospital-observation patients and CT− versus CT+ patients sampled within 24 h. Their specificity, however, was not as high (32% and 27%, respectively, for S100b and GFAP), compared with 6 h after the trauma. HFABP was neutral in these conditions.

TBI, even in mild forms, has a complex physiopathology involving several mechanisms of injuries.^31,32^ First, primary brain injuries are caused by cerebral hemorrhages, skull fractures, focal damages after direct impact on the head, or diffuse axonal damages resulting from the stretching of the brain tissue after a rapid acceleration and deceleration of the brain. Then, biochemical and cellular alterations such as neuroinflammation lead to secondary brain injuries. This suggests that several biomarkers incorporated into a panel could better reflect the injury.^33,34^ In this study, combining GFAP and HFABP increased the specificity above 60%, whereas sensitivity remained at 100%. The clinical impact for the management of patients in an ED becomes, in this study, significant.

This is the first study focusing only on children with mTBI and included also non-CT-scanned patients, to evaluate blood biomarker’s single and combined performances. Pediatric research is crucial as the direct translation of results from adult to children is not obvious. Children’s brain is still in development, and pathological mechanisms involved after injuries and their kinetics might differ from adults. Scandinavian (2003) and French (2023) guidelines have included S100b measurement in the initial management of adult patients suffering from minimal, mild, and moderate TBI.^15–17^ GFAP has also been extensively studied in adults (either alone or in combination with Ubiquitin carboxy-terminal hydrolase L1 (UCHL-1))^19,35,36^ and has been included in the French guidelines.^17^

The World Health Organization set REASSURED criteria for diagnostic tools (Real-time connectivity, Ease of specimen collection, Affordable, Sensitive, Specific, User-friendly, Rapid and robust, Equipment-free, and Deliverable to end-users) and even child-friendly sampling.^37^ Those conditions could be fulfilled using a finger-prick point-of-care testing device (POCT), as it is often used in pediatrics. Two of the three biomarkers tested in our study have already been developed to be used in a POCT for the diagnostic of ICI in adults: the TBICheck^TM^ Rapid test^38^ for HFABP and the i-STAT for GFAP.^36^ The development of these diagnostic devices will be necessary to implement blood biomarker measurement in mTBI clinical routine practice, especially in children. This also raises the need to agree on a validated and standardized technique of measurement, to define applicable cutoff values.

Finally, this study has both considerable strengths and limitations. Our study is constrained by a relatively small sample size. This restricts our ability to conduct further analyses, such as age stratification to investigate cutoff values, selection of patients with blood sampling done only within 3 h, or exploration of combinations of markers to enhance their predictive accuracy. Future investigations could consider additional biomarkers, including those related to blood-based inflammatory markers, which have recently received increased attention in the context of mTBI.^39^ For instance, studies in adults have examined the diagnostic potential of IL10, both independently and in conjunction with markers such as GFAP, HFABP, and S100b.^13^ In the realm of pediatric mTBI, there is a pressing need to collaborate across multiple centers and recruit larger cohorts to advance our understanding.

## Conclusions

In a pediatric mTBI cohort, S100b, GFAP, and HFABP can identify up to 52% of CT− or in-hospital-observation patients while detecting all children with ICI. These markers can significantly help in the management of patients in the ED if sampled within 6 h post-trauma, avoiding unnecessary CT scan, and helping to reduce length of stay for children and their families. Their use in combination can even yield more than 60% of specificity but needs to be confirmed in a larger cohort. These results also highlight the importance to specifically study pediatric patients, as the challenges differ in terms of diagnostic and management workflows compared with the adult population. These findings will require further validation in a larger multicenter cohort before clinical application.

## Supplementary Material

Supplementary Figure S1

## Supplementary Material

Supplementary Figure S2

## Supplementary Material

Supplementary Figure S3

## Supplementary Material

Supplementary Table S1

## Supplementary Material

Supplementary Table S2

## Supplementary Material

Supplementary Table S3

## Supplementary Material

Supplementary Table S4

## Supplementary Material

Supplementary Table S5

## Supplementary Material

Supplementary Table S6

## Supplementary Material

Supplementary Table S7

## Supplementary Material

Supplementary Table S8

## Data Availability

The data presented in this study are available on request from the corresponding author.

## Abbreviations Used

S100b: S100 calcium-binding protein
BGFAP: Glial Fibrillary Acidic Protein
HFABP: Heart Fatty-Acid-Binding Protein
UCHL-1: Ubiquitin Carboxy-terminal Hydrolase L1
IL10: Interleukin 10
TBI: Traumatic Brain Injury
mTBI: mild Traumatic Brain Injury
ICI: Intracranial injury
CT: Computed tomography
GCS: Glasgow Coma Scale
ED: Emergency Department
SP: Specificity
SE: Sensitivity
LOC: Loss of Consciousness
PECARN: Pediatric Emergency Care Applied Research Network
LLoD: Lower Limit of Detection
LLoQ: Lower Limit of Quantification
CV: Coefficient of Variation
ROC: Receiver Operating Characteristic
IQR: Inter Quartile Range
SD: Standard Deviation
Min: Minimum
Max: Maximum
AUC: Area Under the Curve
REASSURED: Real-time connectivity, Ease of specimen collection, Affordable, Sensitive, Specific, User-friendly, Rapid and robust, Equipment-free, and Deliverable to end-users
POCT: Point Of Care Test
HUG: Hopitaux Universitaires de Genève
