# Supplementary material for: Management of Pediatric Mild Traumatic Brain Injury Patients: S100b, Glial Fibrillary Acidic Protein, and Heart Fatty-Acid-Binding Protein Promising Biomarkers
Source: Neurotrauma Rep. 2024 May 31;5(1):529–39. doi: 10.1089/neur.2024.0027 (PMC11271147; doi:10.1089/neur.2024.0027)
Supplement: Supplementary Figure S1 [file neur.2024.0027_chiollaz_supplemental.docx]

**SUPPLEMENTAL DATA**

Title: **Management of paediatric mild traumatic brain injury patients: S100b, GFAP and HFABP promising biomarkers.**

**- TABLES:**

**Table 1. Suppl:** Biomarkers expression in CT-scanned mTBI patients (within 6 hours).

**Table 2. Suppl:** Biomarkers expression in CT-scanned and in-hospital-observation mTBI patients (within 6 hours).

**Table 3. Suppl:** Panels best performances to rule-out mTBI patients (within 6 hours).

**table 4. Suppl:** Clinical parameters and biomarkers expression in controls and mTBI patients (with or without CT scan) (within 24 hours).

**Table 5. Suppl:** PECARN criteria for positive CT (within 24 hours).

**Table 6. Suppl:** Biomarkers expression in CT-scanned mTBI patients (within 24 hours).

**Table 7. Suppl:** Biomarkers expression in CT-scanned and in-hospital-observation mTBI patients (within 24 hours).

**Table 8. Suppl:** S100b, GFAP, and HFABP best performances to rule-out mTBI patients (within 24hours).

**- FIGURES:**

**Figure1. Suppl:** Biomarkers and age correlation in controls patient (n=74).

**Figure 2. Suppl:** S100b, GFAP, and HFABP serum concentration in mTBI patients regarding their CT scan result (within 24 hours).

**Figure 3. Suppl:** S100b, GFAP, and HFABP diagnostic performances to classify mTBI patients (within 24hours).

**Table 1. Suppl:** Biomarkers expression in CT-scanned mTBI patients (within 6 hours).

|  | **CT- (N=36)** | **CT+ (N=7)** | **P-value** |
| --- | --- | --- | --- |
| **S100b (pg/ml)** |  |  |  |
| Mean (SD) | 92.4 (80.5) | 191 (198) | 0.186 |
| Median [Min, Max] | 75.3 [10.1, 316] | 89.9 [44.4, 583] |  |
| Missing | 4 (11.1%) | 0 (0%) |  |
| **GFAP (pg/ml)** |  |  |  |
| Mean (SD) | 737 (967) | 1820 (2460) | 0.084 |
| Median [Min, Max] | 354 [13.2, 4690] | 771 [215, 7120] |  |
| **HFABP (pg/ml)** |  |  |  |
| Mean (SD) | 4710 (5280) | 5470 (3960) | 0.335 |
| Median [Min, Max] | 3190 [1080, 30400] | 3600 [2470, 12600] |  |
| Missing | 1 (2.8%) | 0 (0%) |  |

**Table 2. Suppl:** Biomarkers expression in CT-scanned and In-hospital observation mTBI patients (within 6 hours).

|  | **CT- or Observation (N=215)** | **CT+ (N=7)** | **P-value** |
| --- | --- | --- | --- |
| **S100b (pg/ml)** |  |  |  |
| Mean (SD) | 71.3 (75.6) | 191 (198) | 0.031 |
| Median [Min, Max] | 51.8 [1.19, 672] | 89.9 [44.4, 583] |  |
| Missing | 18 (8.4%) | 0 (0%) |  |
| **GFAP (pg/ml)** |  |  |  |
| Mean (SD) | 516 (740) | 1820 (2460) | 0.008 |
| Median [Min, Max] | 208 [0.787, 4690] | 771 [215, 7120] |  |
| Missing | 2 (0.9%) | 0 (0%) |  |
| **HFABP (pg/ml)** |  |  |  |
| Mean (SD) | 3640 (3220) | 5470 (3960) | 0.07 |
| Median [Min, Max] | 2690 [1080, 30400] | 3600 [2470, 12600] |  |
| Missing | 5 (2.3%) | 0 (0%) |  |

**Table 3. Suppl**: Panels best performances to rule-out mTBI patients (within 6 hours)

**(A)**

| **Panels of biomarkers** | **Sensitivity (%)** | **Specificity (%)** | **Threshold (pg/ml)** |
| --- | --- | --- | --- |
| GFAP- HFABP | 100 | 62.86 | 204.22 ; 2456.57 |
| GFAP-S100b | 100 | 59.38 | 204.22 ; 43.06 |
| S100b-HFABP | 100 | 48.39 | 43.06 ; 2456.57 |

**(B)**

| **Panels of biomarkers** | **Sensitivity (%)** | **Specificity (%)** | **Threshold (pg/ml)** |
| --- | --- | --- | --- |
| GFAP- HFABP | 100 | 67.94 | 214.02 ; 2456.57 |
| GFAP-S100b | 100 | 66.33 | 214.02 ; 44.34 |
| S100b-HFABP | 100 | 57.51 | 44.34 ; 2456.57 |

1. **Best performances to rule-out a maximum of CT-negative patients, while all CT-positive patients have been identified** (only CT-scanned patients).
2. **Best performances to rule-out a maximum CT-negative and in-hospital-observation patients, while all CT-positive patients have been identified** (CT-scanned and observed without CT [>6hours at ED] patients).

**table 4. Suppl:** Clinical parameters and biomarkers expression in controls and mTBI patients (with or without CT scan) (within 24 hours).

|  | |  | | **mild TBI**  **N=302** | | | | | |  | |
| --- | --- | --- | --- | --- | --- | --- | --- | --- | --- | --- | --- |
|  | | **Ctrl N=74** | | **no CT**  **N=230 (*76%)*** | | **CT**  **N=72 (*24%)*** | | | |  | |
|  | |  | |  | | **CT- N=54 (*75%)*** | | **CT+ N=18 (*25%)***  ***(6% of mTBI)*** | | **P-value** | |
| **Age (yo)** | |  | |  | |  | |  | |  | |
| Mean (SD) | | 8.70 (4.93) | | 8.58 (4.43) | | 8.28 (4.78) | | 8.04 (4.85) | | 0.941 | |
| Median [Min, Max] | | 8.75 [0.10, 16.8] | | 9.05 [0.20, 16.0] | | 8.00 [0.10, 16.0] | | 8.20 [0.11, 15.0] | |  | |
| **Sex** | |  | |  | |  | |  | |  | |
| Boys | | 40 (54.1%) | | 129 (56.1%) | | 33 (61.1%) | | 13 (72.2%) | | 0.491 | |
| Girls | | 34 (45.9%) | | 101 (43.9%) | | 21 (38.9%) | | 5 (27.8%) | |  | |
| **Severity of injury, n (%)** | |  | |  | |  | |  | |  | |
| GCS14 | | - | | 16 (7.0%) | | 13 (24.1%) | | 4 (22.2%) | | <0.001 | |
| GCS15 | | - | | 214 (93.0%) | | 41 (75.9%) | | 14 (77.8%) | |  | |
| **Symptoms at inclusion, n (%)** | |  | |  | |  | |  | |  | |
| Loss of consciouness | | - | | 46 (20.0%) | | 10 (18.5%) | | 1 (5.6%) | | 0.382 | |
| Post-traumatic amnesia | | - | | 71 (30.9%) | | 14 (25.9%) | | 1 (5.6%) | | 0.117 | |
| Persistent headaches | | - | | 72 (31.3%) | | 12 (22.2%) | | 7 (38.9%) | | 0.367 | |
| More than 3 vomit's episods | | - | | 44 (19.1%) | | 9 (16.7%) | | 6 (33.3%) | | 0.155 | |
| Vertigo | | - | | 21 (9.1%) | | 2 (3.7%) | | 1 (5.6%) | | 0.565 | |
| Confusion | | - | | 27 (11.7%) | | 10 (18.5%) | | 1 (5.6%) | | 0.095 | |
| Convulsion | | - | | 3 (1.3%) | | 2 (3.7%) | | 0 (0%) | | 0.271 | |
| **TBI and others body fractures, n (%)** | |  | |  | |  | |  | |  | |
| TBI only | - | | 218 (94.8%) | | 46 (85.2%) | | 15 (83.3%) | | 0.004 | |  |
| TBI + others fractures | - | | 9 (3.9%) | | 8 (14.8%) | | 3 (16.7%) | |  | |  |
| **Skull fracture (on CT) , n (%)** |  | |  | |  | |  | |  | |  |
| yes | - | | - | | 12 (22.2%) | | 15 (83.3%) | | <0.001 | |  |
| **timelaps TBI-blood (hours)** |  | |  | |  | |  | |  | |  |
| Mean (SD) | - | | 6.06 (4.25) | | 7.03 (5.83) | | 9.91 (7.36) | | 0.103 | |  |
| Median [Min, Max] | - | | 5.00 [1.00, 23.0] | | 5.00 [1.00, 24.0] | | 8.00 [2.00, 24.0] | |  | |  |
| Missing | - | | 2 (0.9%) | | 1 (1.9%) | | 0 (0%) | |  | |  |
| **S100b (pg/ml)** |  | |  | |  | |  | |  | |  |
| Mean (SD) | 39.3 (38.7) | | 64.8 (68.6) | | 80.8 (73.4) | | 127 (142) | | <0.001 | |  |
| Median [Min, Max] | 27.9 [1.79, 220] | | 51.0 [0.171, 672] | | 52.1 [3.39, 316] | | 74.6 [37.2, 583] | |  | |  |
| Missing | 11 (14.9%) | | 20 (8.7%) | | 7 (13.0%) | | 2 (11.1%) | |  | |  |
| **GFAP (pg/ml)** |  | |  | |  | |  | |  | |  |
| Mean (SD) | 97.6 (141) | | 469 (737) | | 681 (936) | | 1770 (1970) | | <0.001 | |  |
| Median [Min, Max] | 64.0 [3.37, 951] | | 195 [0.787, 4830] | | 276 [11.5, 4690] | | 847 [79.8, 7120] | |  | |  |
| Missing | 4 (5.4%) | | 2 (0.9%) | | 0 (0%) | | 0 (0%) | |  | |  |
| **HFABP (pg/ml)** |  | |  | |  | |  | |  | |  |
| Mean (SD) | 2270 (3830) | | 3120 (2380) | | 4030 (4800) | | 3570 (3010) | | <0.001 | |  |
| Median [Min, Max] | 1580 [790, 33600] | | 2480 [919, 17900] | | 2530 [943, 30400] | | 2790 [980, 12600] | |  | |  |
| Missing | 1 (1.4%) | | 5 (2.2%) | | 2 (3.7%) | | 0 (0%) | |  | |  |

**Table 5. Suppl :** PECARN criteria for positive CT (within 24hours).

|  | **CT+ (N=18)** |
| --- | --- |
| **1.Intracranial hemorrhage or contusion** | 15 (83.3%) |
| **-sub-arachnoidal hemorrhage** | 1 (5.6%) |
| **-epidural hemorrhage** | 2 (11.1%) |
| **-intra-parenchymal hemorrhage** | 2 (11.1%) |
| **-sub-dural hemorrhage** | 13 (72.2%) |
| **2.Cerebral oedema** | 0 (0%) |
| **3.Traumatic infraction** | 0 (0%) |
| **4.Diffuse axonal injury or shearing injury** | 0 (0%) |
| **5.Sigmoïd sinus thrombosis** | 0 (0%) |
| **6.Midline shift of intracranial contents or signs of brain herniation** | 1 (5.6%) |
| **7.Diastasis of the skull** | 6 (33.3%) |
| **8.Pneumocephalus** | 9 (50.0%) |
| **9.Skull fracture depressed by at least the width of the table of the skull** | 3 (16.7%) |

**Table 6. Suppl:** Biomarkers expression in CT-scanned mTBI patients (within 24 hours).

|  | **CT- (N=54)** | **CT+ (N=18)** | **P-value** |
| --- | --- | --- | --- |
| **S100b (pg/ml)** |  |  |  |
| Mean (SD) | 80.8 (73.4) | 127 (142) | 0.101 |
| Median [Min, Max] | 52.1 [3.39, 316] | 74.6 [37.2, 583] |  |
| Missing | 7 (13.0%) | 2 (11.1%) |  |
| **GFAP (pg/ml)** |  |  |  |
| Mean (SD) | 681 (936) | 1770 (1970) | 0.006 |
| Median [Min, Max] | 276 [11.5, 4690] | 847 [79.8, 7120] |  |
| **HFABP (pg/ml)** |  |  |  |
| Mean (SD) | 4030 (4800) | 3570 (3010) | 0.989 |
| Median [Min, Max] | 2530 [943, 30400] | 2790 [980, 12600] |  |
| Missing | 2 (3.7%) | 0 (0%) |  |

**Table 7. Suppl:** Biomarkers expression in CT-scanned and in-hospital-observation mTBI patients (within 24 hours).

|  | **CT- or Observation (N=284)** | **CT+ (N=18)** | **P-value** |
| --- | --- | --- | --- |
| **S100b (pg/ml)** |  |  |  |
| Mean (SD) | 67.7 (69.7) | 127 (142) | 0.011 |
| Median [Min, Max] | 51.0 [0.171, 672] | 74.6 [37.2, 583] |  |
| Missing | 27 (9.5%) | 2 (11.1%) |  |
| **GFAP (pg/ml)** |  |  |  |
| Mean (SD) | 510 (782) | 1770 (1970) | <0.001 |
| Median [Min, Max] | 196 [0.787, 4830] | 847 [79.8, 7120] |  |
| Missing | 2 (0.7%) | 0 (0%) |  |
| **HFABP (pg/ml)** |  |  |  |
| Mean (SD) | 3290 (2990) | 3570 (3010) | 0.707 |
| Median [Min, Max] | 2480 [919, 30400] | 2790 [980, 12600] |  |
| Missing | 7 (2.5%) | 0 (0%) |  |

**Table 8. Suppl:** S100b, GFAP, and HFABP best performances to rule-out mTBI patients (within 24hours).

**(A)**

| **Biomarker** | **Sensitivity (%)** | **Specificity (%)** | **AUC (%)** | **Threshold (pg/ml)** |
| --- | --- | --- | --- | --- |
| S100b | 100 | 31.91 | 63.83 | 36.32 |
| GFAP | 100 | 22.22 | 71.60 | 78.38 |
| HFABP | 100 | 1.92 | 50.11 | 961.80 |

**(B)**

| **Biomarker** | **Sensitivity (%)** | **Specificity (%)** | **AUC (%)** | **Threshold (pg/ml)** |
| --- | --- | --- | --- | --- |
| S100b | 100 | 31.52 | 68.90 | 37.13 |
| GFAP | 100 | 27.30 | 77.32 | 79.65 |
| HFABP | 100 | 1.44 | 52.65 | 979.49 |

1. **Best performances to rule-out a maximum of CT-negative patients, while all CT-positive patients have been identified** (only CT-scanned patients).
2. **Best performances to rule-out a maximum CT-negative and in-hospital-observation patients, while all CT-positive patients have been identified** (CT-scanned and observed without CT [>6hours at ED] patients).

**Figure1. Suppl:** Biomarkers and age correlation in controls patient (n=74).

**
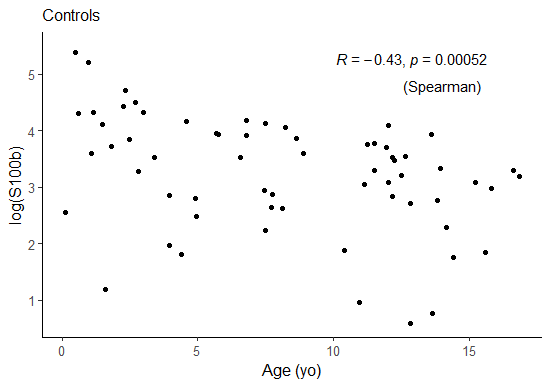

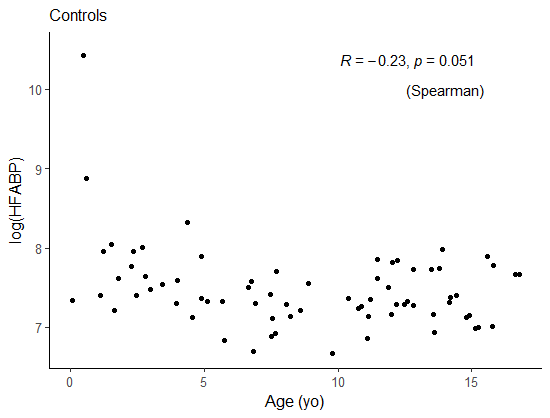

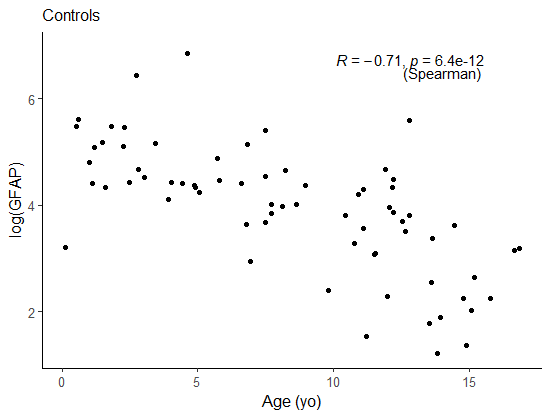
**A)

**
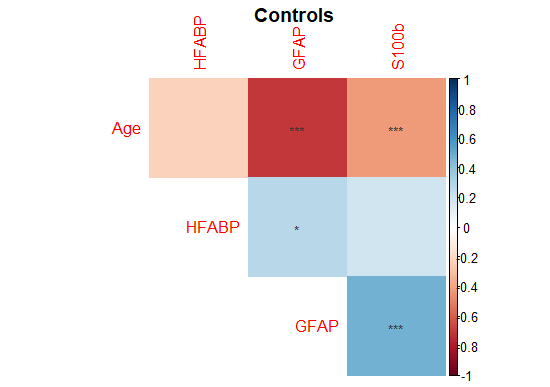
**

B)

1. Log scaled biomarker’s concentration of S100b, GFAP and HFABP in function of controls’ age (years old). R =Spearman correlation coefficient, p= Pvalue.
2. Log scaled biomarker’s concentration of S100b, GFAP and HFABP in function of controls’ age (years old). R =Spearman correlation coefficient, p= Pvalue. Spearman correlation matrix. ***Pvalue <0.0001, * Pvalue <0.05

**Figure 2. Suppl :** S100b, GFAP, and HFABP serum concentration in mTBI patients regarding their CT scan result (within 24 hours).

**(A)
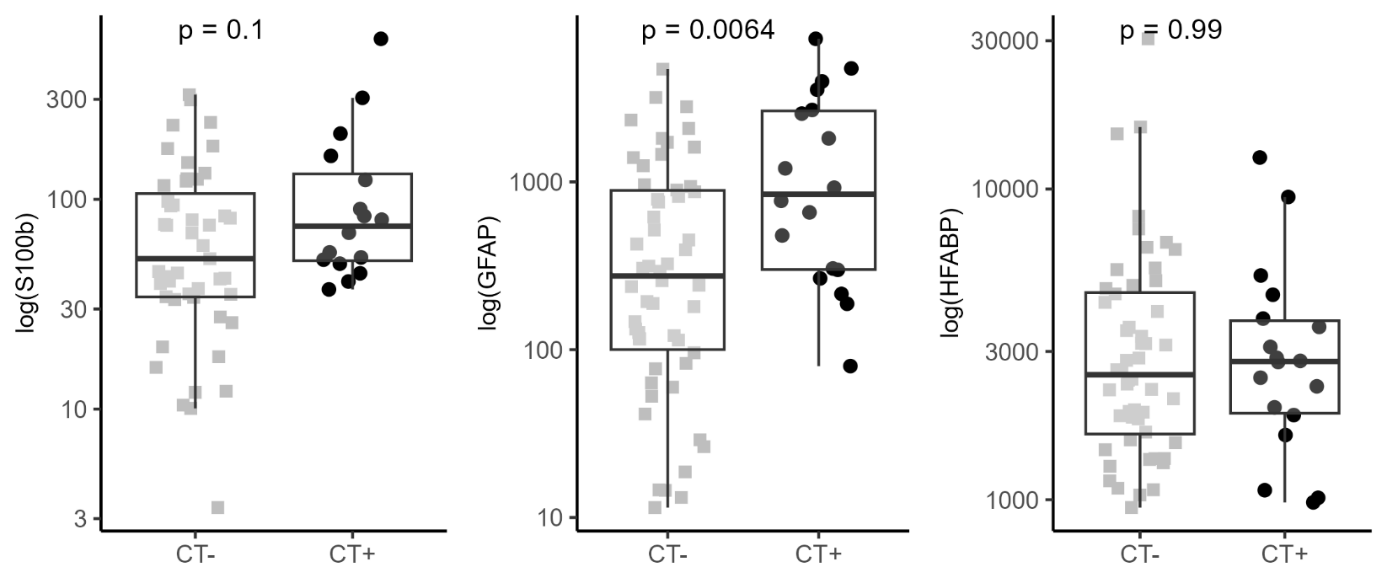
**

**(B)**
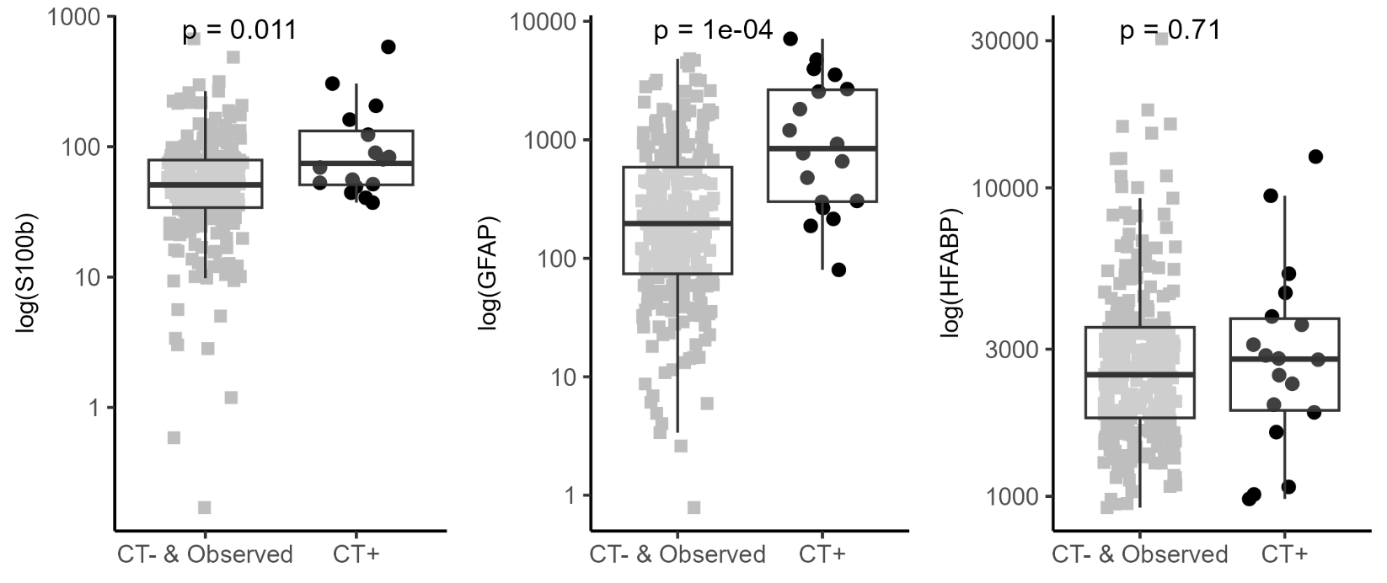


1. **Biomarkers expression within CT-negative and CT-positive mTBI patients** (only CT-scanned patients).
2. **Biomarkers expression within CT-negative or in-hospital-observation patients and CT-positive mTBI patients** (CT-scanned and observed without CT [>6hours at ED] patients).

Positive CT is based on PECARN criteria. Box plots represent median and IQR for compared groups; dot plots represent for each patient log scaled biomarker’s concentration. The analysis was carried out using a Mann-Whitney U test (shown p-value).

**Figure 3. Suppl:** S100b, GFAP, and HFABP diagnostic performances to classify mTBI patients (within 24hours).

**
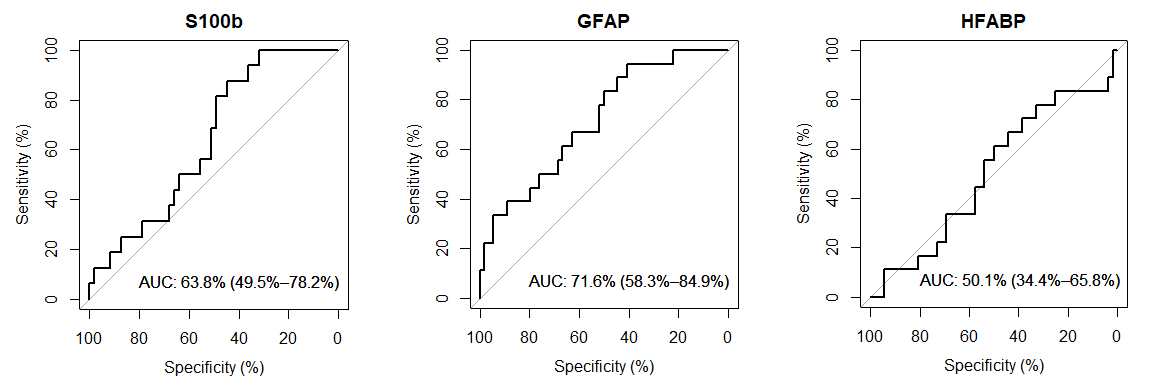
(A)**

**
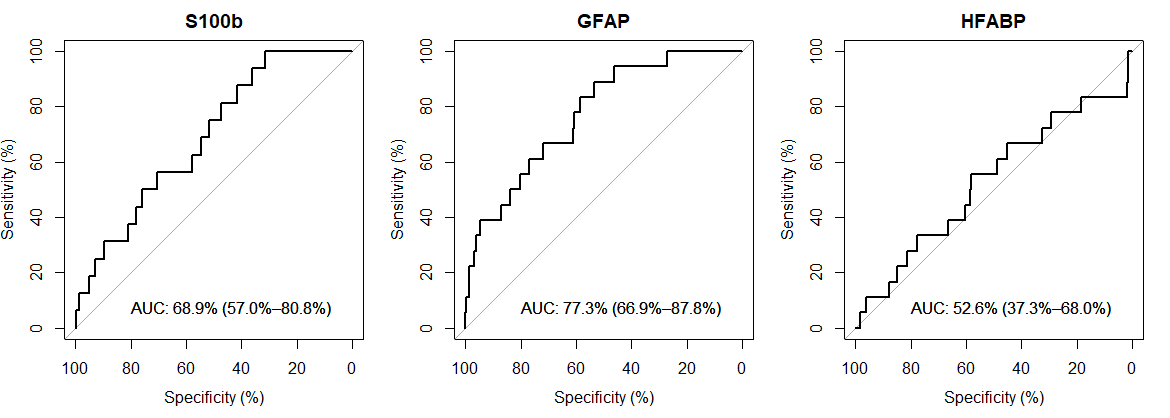
(B)**

1. **Diagnostic performances within CT-negative and CT-positive mTBI patients** (only CT-scanned patients).
2. **Diagnostic performances within CT-negative or in-hospital-observation patients and CT-positive mTBI patients** (CT-scanned and observed without CT [>6hours at ED] patients).

Receiver Operating Characteristic (ROC) Curves in mTBI patients. AUC= Area Under the Curve with 95% confidence interval. Performances were investigated at 100% sensitivity and corresponding highest specificity.
